# Evaluation of EMLA Cream for Preventing Pain during Tattooing of Rabbits: Changes in Physiological, Behavioural and Facial Expression Responses

**DOI:** 10.1371/journal.pone.0044437

**Published:** 2012-09-07

**Authors:** Stephanie C. J. Keating, Aurelie A. Thomas, Paul A. Flecknell, Matthew C. Leach

**Affiliations:** 1 Department of Clinical Studies, Ontario Veterinary College, University of Guelph, Guelph, Ontario, Canada; 2 Institute of Neuroscience and Comparative Biology Centre, Newcastle University, Newcastle upon Tyne, United Kingdom; Université Pierre et Marie Curie, France

## Abstract

**Background:**

Ear tattooing is a routine procedure performed on laboratory, commercial and companion rabbits for the purpose of identification. Although this procedure is potentially painful, it is usually performed without the provision of analgesia, so compromising animal welfare. Furthermore, current means to assess pain in rabbits are poor and more reliable methods are required. The objectives of this study were to assess the physiological and behavioural effects of ear tattooing on rabbits, evaluate the analgesic efficacy of topical local anaesthetic cream application prior to this procedure, and to develop a scale to assess pain in rabbits based on changes in facial expression.

**Methodology/Principal Findings:**

In a crossover study, eight New Zealand White rabbits each underwent four different treatments of actual or sham ear tattooing, with and without prior application of a topical local anaesthetic (lidocaine/prilocaine). Changes in immediate behaviour, heart rate, arterial blood pressure, serum corticosterone concentrations, facial expression and home pen behaviours were assessed. Changes in facial expression were examined to develop the Rabbit Grimace Scale in order to assess acute pain. Tattooing without EMLA cream resulted in significantly greater struggling behaviour and vocalisation, greater facial expression scores of pain, higher peak heart rate, as well as higher systolic and mean arterial blood pressure compared to all other treatments. Physiological and behavioural changes following tattooing with EMLA cream were similar to those in animals receiving sham tattoos with or without EMLA cream. Behavioural changes 1 hour post-treatment were minimal with no pain behaviours identifiable in any group. Serum corticosterone responses did not differ between sham and tattoo treatments.

**Conclusions:**

Ear tattooing causes transient and potentially severe pain in rabbits, which is almost completely prevented by prior application of local anaesthetic cream. The Rabbit Grimace Scale developed appears to be a reliable and accurate way to assess acute pain in rabbits.

## Introduction

The marking of animals for identification is one of the most common procedures carried out on laboratory, farm and some pet animal species. Although a wide range of methods are available and routinely used we know little about the effect of these on the animals being marked. Being able to clearly identify individuals is a fundamental and often a legal requirement for many species, particularly for those undergoing treatments or procedures, breeding animals, and those being transported, sold or shown. While a number of identification methods are available for use in rabbits [1], ear tattooing is routinely used in farm, laboratory and some pet rabbits, as it is considered rapid and cost-effective, and provides a permanent identification. While tattooing has clear benefits, the procedure causes significant cardiovascular changes in rats, which may be indicative of pain [2]. Thus, the practice of ear tattooing without the provision of adequate analgesia poses welfare concerns and general anaesthesia has been recommended to prevent the immediate pain of tattoo administration in laboratory animals [3]. Although effective, it is preferable to avoid general anaesthesia as it carries a significant risk, particularly in rabbits and small mammals [4]. It is also not normally possible for non-veterinarians to acquire and administer general anaesthetic agents. It is therefore commonplace for ear tattooing of rabbits to be carried out with no anaesthesia or analgesia [5].

EMLA (Eutectic Mixture of Local Anesthetics) cream is a eutectic mixture of 2.5% prilocaine and 2.5% lidocaine that can easily be applied to provide topical anaesthesia. The use of EMLA cream has been used in a range of species to reduce or prevent pain associated with intravenous and arterial catheter placement, vaccination, biopsies, skin graft removal, and even minor surgical procedures with minimal side effects [6]–[10]. EMLA has not yet been evaluated for use in ear tattoo administration; however, if effective it would provide an easy, safe and accessible means to ensure adequate analgesia in rabbits undergoing ear tattooing. It is also available without prescription in both the UK and North America.

In order to evaluate both the potential negative effects associated with tattooing and the positive influence of EMLA administration, we must be able to recognise pain effectively and assess its severity. Unfortunately, validated methods of assessing the pain associated with tattooing and other routine husbandry and veterinary procedures are lacking for many species including rabbits and our ability to recognise pain is often poor [11]–[13]. Established methods of pain detection have limited potential for the clinical assessment of spontaneous acute pain. For example, evoked responses, such as those induced by the Hargreaves and Von Frey assays, are reflexive measures of nociception and primarily measure hypersensitivity associated with pain [14]. Operant measures are non-reflexive and evaluate the conscious pain state [15], but are impractical to perform for the assessment of acute pain such as that associated with tattooing. Currently used measures of spontaneous pain tend to rely on either subjective assessment of ‘clinical signs’ (e.g. appearance, vocalisations and demeanour) or more objective assessments of food and water consumption and body weight etc. Although, these are clinically applicable they are often non-specific, retrospective in nature and provide little evidence as to how the changes observed indicate pain intensity [16], [17]. Behaviour-based pain scoring systems are considered to overcome these limitations and have been successfully developed for rabbits [17]. However they are not without their own limitations, in particular they require extensive observation time and this may limit their application, and have only been developed for a small number of procedures in a limited range of species [18].

The inherent limitations of conventional pain assessment methods have prompted the search for reliable cage-side indicators of pain. Changes in facial expression have recently been evaluated in the mouse and rat and have proven to be an accurate, repeatable and valid way to identify pain in these species [18]–[20] that overcomes some of the limitations of existing measures. The rodent pain face shares common features with that of humans suggesting evolutionary conservation of key expressions [21]. If this is the case, then it is possible that other mammalian species, such as rabbits, exhibit similar facial changes when experiencing pain.

The objectives of the current study were to assess the physiologic and behavioural impact of ear tattooing on rabbits, evaluate the analgesic efficacy of EMLA cream during this procedure and to develop a pain scale based on facial expressions in rabbits.

## Results

Treatment and time were the only factors to significantly influence the parameters measured. Treatment order had no significant influence on any measure.

### Immediate Reaction to the Tattooing Procedure

No vocalisation or struggling was observed when the animals underwent the sham tattooing with or without prior application of EMLA. One rabbit in the group that was tattooed after application of EMLA exhibited vocalisation but no struggling was observed in any animals in this group. Considerable and significantly greater struggling and vocalisation was observed in animals tattooed without prior application of EMLA (P = 0.000), with all animals in this group vocalising and struggling violently to escape restraint for up to 10 seconds during the tattoo procedure.

### Cardiovascular Responses

#### Heart rate

**Figure 1:**
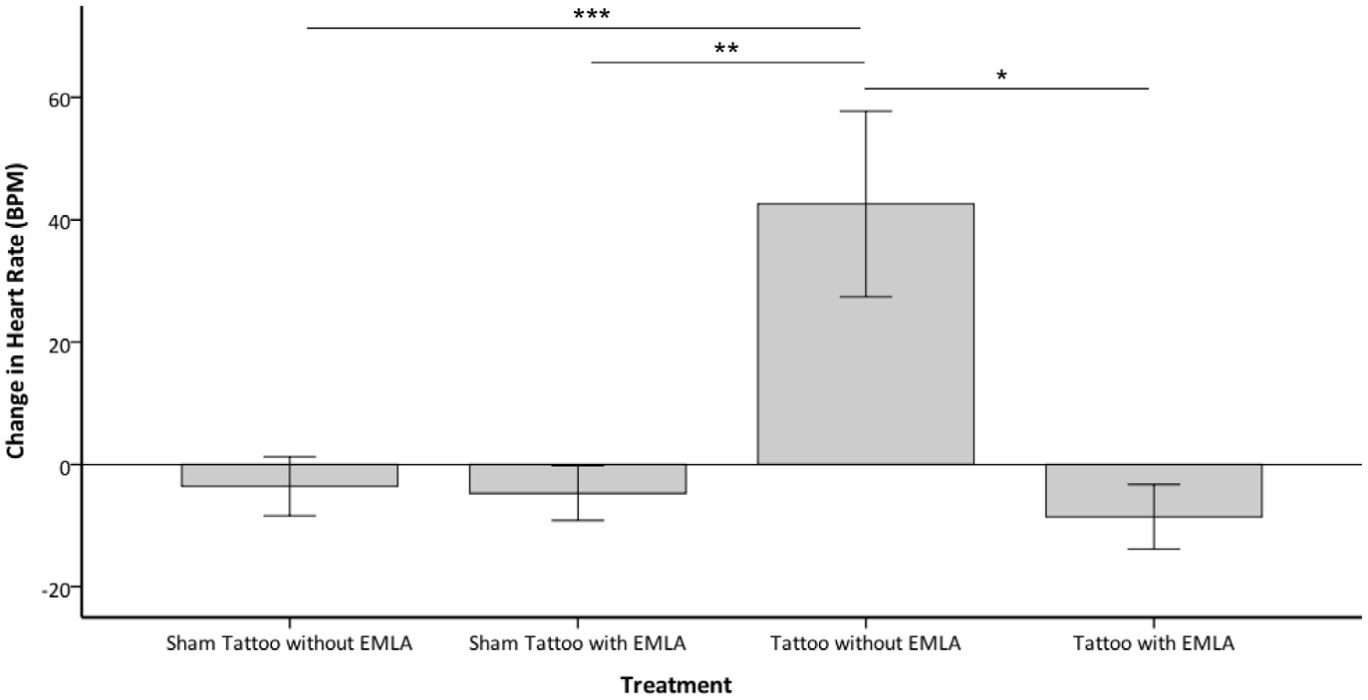
Change in mean heart rate from pre to post-procedure. Change in mean heart rate (beats per minute ±1 SE) from pre to post-procedure observed in rabbits (y-axis) when undergoing the various treatments (x-axis); sham tattooing with and without prior application of EMLA and tattooing with and without prior application of EMLA (n = 8 per treatment) (^★^P = 0.022, ^★★^P = 0.031, ^★★★^P = 0.004).

Treatment had a significant effect on the pre to post procedure change in peak heart rate (P = 0.002). A significantly greater increase from pre to post procedure was observed with tattooing without prior application of EMLA compared to tattooing with prior application of EMLA and sham tattooing with and without prior application of EMLA (P = 0.022, P = 0.031, P = 0.004 respectively), with no further differences between the treatments (see Figure 1). Treatment did not have a significantly effect on the pre to post procedure change in lowest heart rate or area-under-the-curve for heart rate.

#### Systolic arterial pressure (SAP)

**Figure 2:**
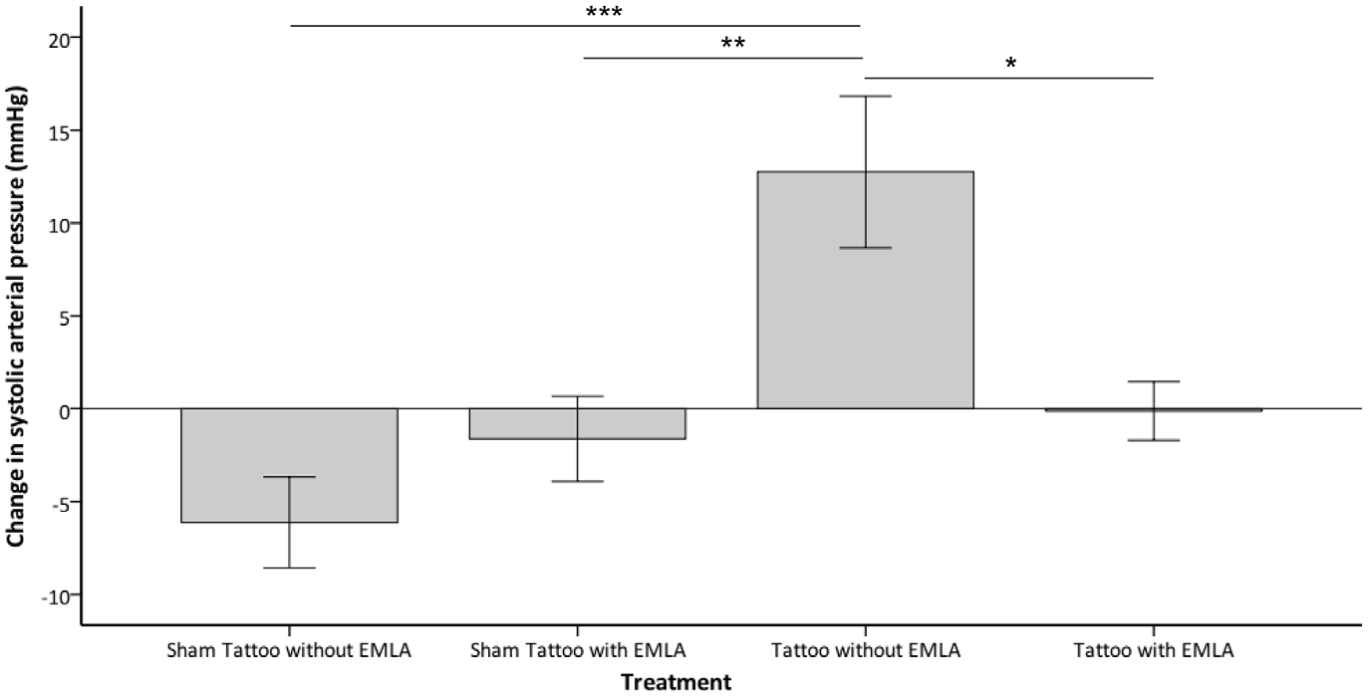
Change in mean systolic arterial pressure (SAP) from pre to post-procedure. Change in mean systolic arterial pressure (mmHg ±1 SE) from pre to post-procedure observed in rabbits (y-axis) when undergoing the various treatments (x-axis); sham tattooing with and without prior application of EMLA and tattooing with and without prior application of EMLA (n = 8 per treatment) (^★^P = 0.024, ^★★^P = 0.028, ^★★★^P = 0.003).

Treatment had a significant effect on the pre to post procedure change in peak SAP (P = 0.001). A significantly greater increase from pre to post procedure was observed with tattooing without prior application of EMLA compared to tattooing with prior application of EMLA and sham tattooing with and without prior application of EMLA (P = 0.024, P = 0.028, P = 0.003 respectively), with no further significant differences between the treatments (see Figure 2). Treatment did not significantly effect the pre to post procedure change in lowest SAP or area-under-the-curve for SAP.

#### Mean arterial pressure (MAP)

**Figure 3:**
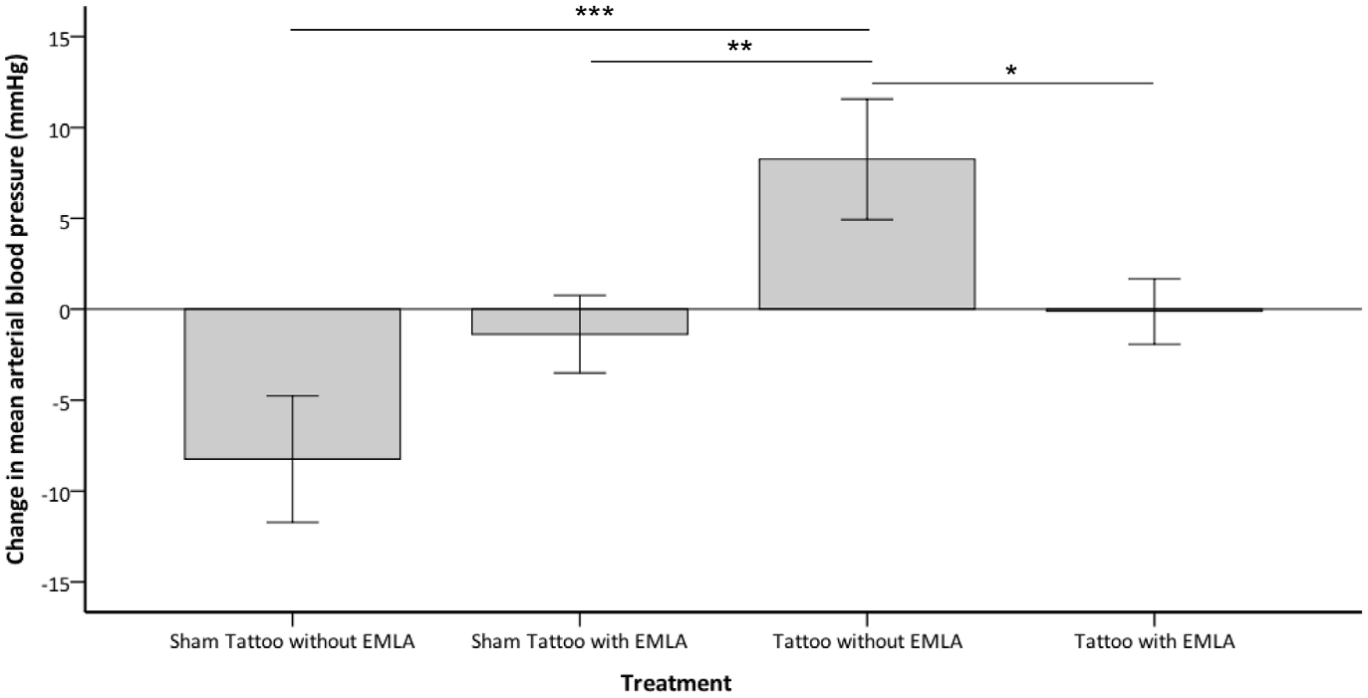
Change in mean arterial pressure (MAP) from pre to post-procedure. Change in mean arterial pressure (mmHg ±1SE) from pre to post-procedure observed in rabbits (y-axis) when undergoing the various treatments (x-axis); sham tattooing with and without prior application of EMLA and tattooing with and without prior application of EMLA (n = 8 per treatment) (^★^P = 0.05, ^★★^P = 0.035, ^★★★^P = 0.01).

Treatment had a significant effect on the pre to post procedure change in peak MAP (P = 0.004). A significantly greater increase from pre to post procedure was observed with tattooing without prior application of EMLA compared to tattooing with prior application of EMLA and sham tattooing with and without prior application of EMLA (P = 0.05, P = 0.035, P = 0.01 respectively), with no further significant differences between the treatments (see Figure 3). Treatment did not significantly effect the pre to post procedure change in lowest MAP or area-under-the-curve for MAP.

#### Diastolic arterial pressure (DAP)

Treatment did not have a significant effect on the pre to post procedure change in peak, lowest or area-under-the-curve for DAP.

### Serum Corticosterone Concentration

**Figure 4:**
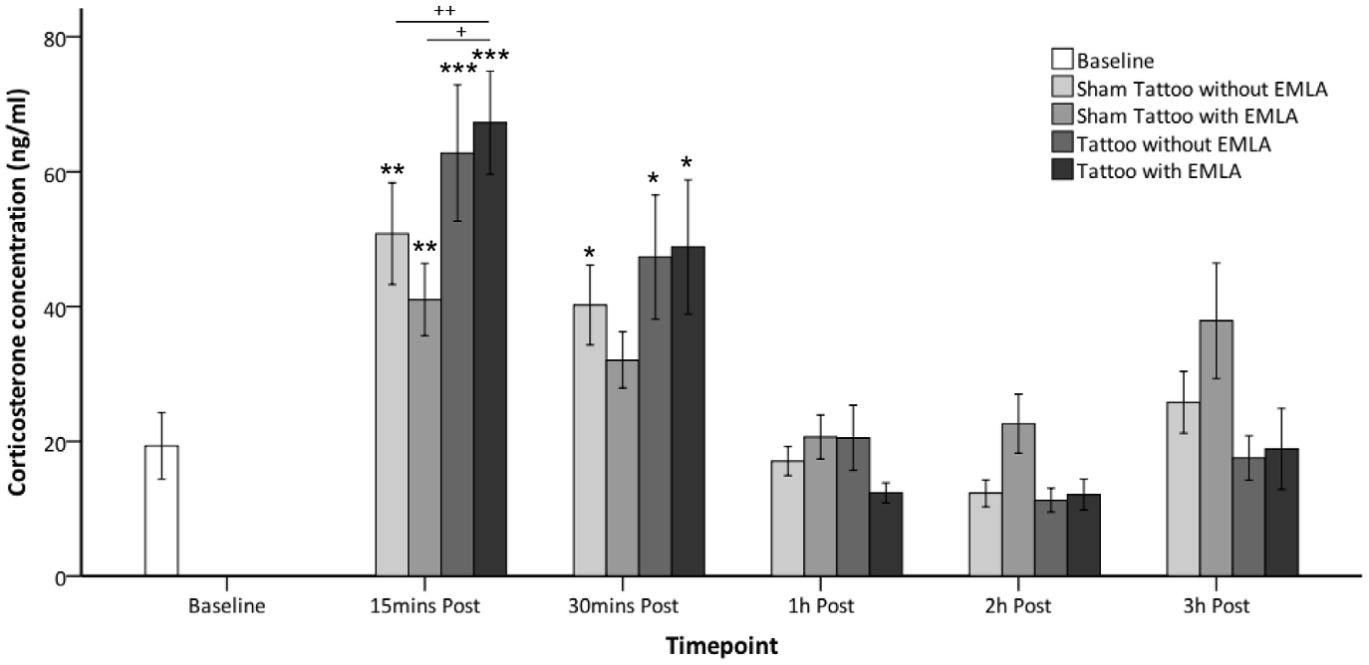
Mean serum corticosterone concentration from pre to post-procedure. Mean corticosterone concentration (ng/ml ±1SE) observed in rabbits (y-axis) at baseline and various post-procedure time points (x-axis) when undergoing the various treatments; sham tattooing with and without prior application of EMLA and tattooing with and without prior application of EMLA (n = 8 per treatment) (Comparison to baseline: ^★^P<0.05, ^★★^P<0.01, ^★★★^P<0.001, Comparison between treatments: ^+^P = 0.05, ^++^P = 0.01).

Time and a time*treatment interaction had significant effects on corticosterone concentration (P<0.001, P<0.001 respectively). Treatment alone did not significantly influence corticosterone concentration. The corticosterone concentration was significantly higher compared to baseline at 15 and 30 minutes post-procedure for sham tattooing without prior application of EMLA (P = 0.002, P = 0.02 respectively), tattooing without prior application of EMLA (P = 0.001, P = 0.02 respectively) and tattooing with prior application of EMLA (P = 0.000, P = 0.02 respectively). For sham tattooing with prior application of EMLA corticosterone concentration was significantly higher compared to baseline at 15 minutes post-procedure (P = 0.01) but not at 30 minutes post procedure. Corticosterone concentration was not significantly different to baseline at 1 hour, 2 hours or 3 hours post-procedure for any of the treatment groups (see Figure 4).

The change in corticosterone concentration from baseline was significantly influenced by treatment at 15 minutes post-procedure (P = 0.05), but not at 30 minutes, 1 hour, 2 hours or 3 hours post-procedure. At 15 minutes post-procedure, the change in corticosterone concentration from baseline was significantly greater for tattooing with and without prior application of EMLA compared to sham tattoo with prior application of EMLA (P = 0.05, P = 0.01 respectively), with no significant differences between the remaining treatments. For area-under-curve (AUC) there was no significant effect of treatment (P = 0.81).

### Rabbit Grimace Scale (RbtGS)

**Figure 5:**
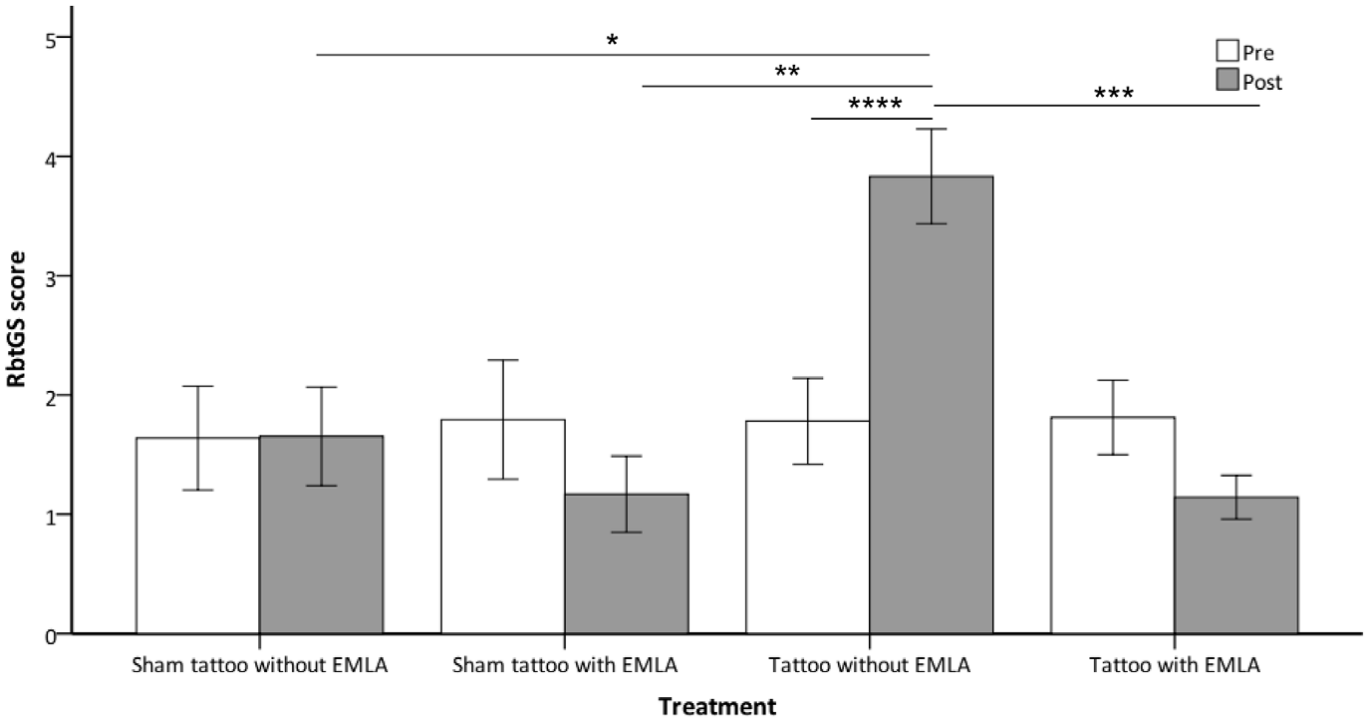
Mean Rabbit Grimace Scale scores pre and intra procedures. Mean RbtGS scores (±1SE) observed in rabbits (y-axis) at baseline and intra-procedure (x-axis) when undergoing the various treatments; sham tattooing with and without prior application of EMLA and tattooing with and without prior application of EMLA (n = 8 per treatment) (^★^P = 0.018, ^★★^P = 0.002, ^★★★^P = 0.001, ^★★★★^P = 0.000).

**Figure 6:**
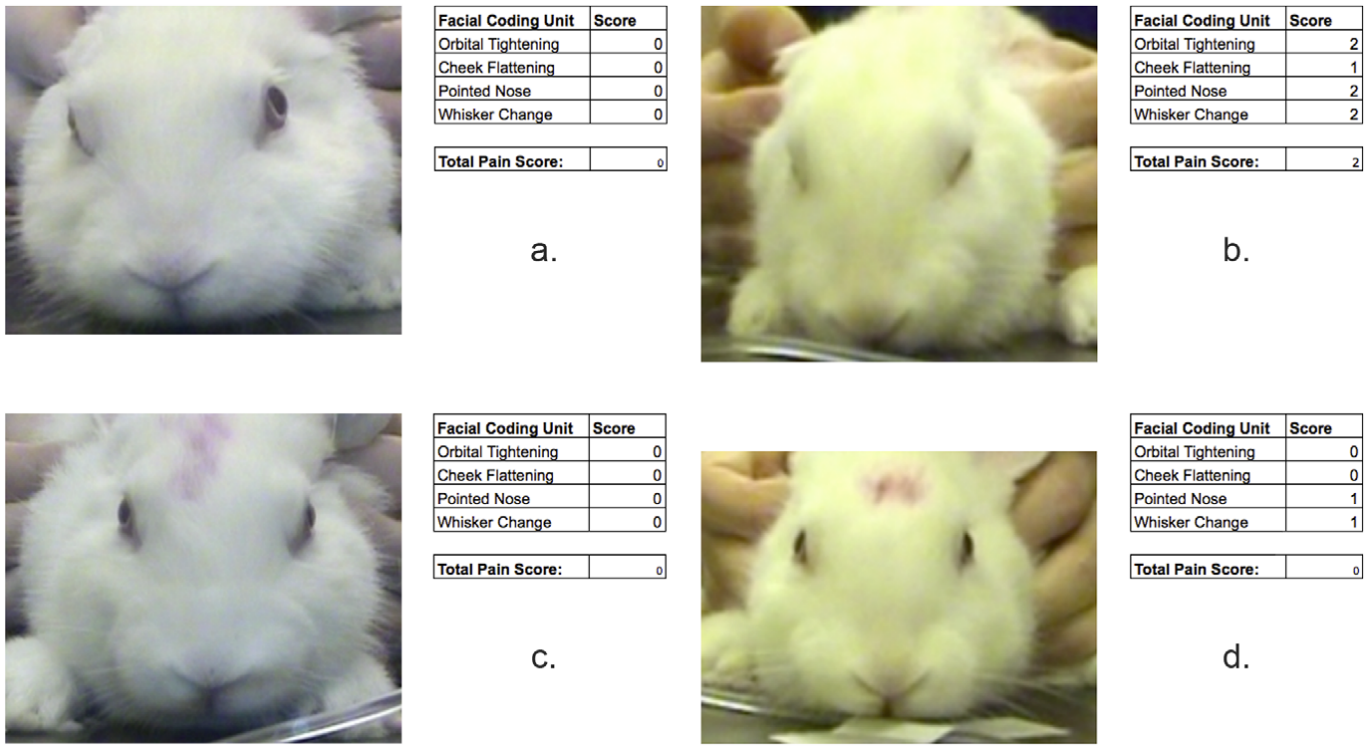
Example images and RbtGs scores. Example images and associated RbtGS scores of a rabbit in the pre-tattoo (a) and intra-tattoo (b) periods without prior application of EMLA and of a rabbit in the pre-sham tattoo (c) and intra-sham tattoo (d) periods without prior application of EMLA.

Treatment and a time*treatment interaction had significant effects on RbtGS score (P = 0.004, P = 0.001, respectively). Time alone did not significantly influence RbtGS score. There was no significant difference between the treatments in the pre-procedure period. Intra-procedure, RbtGS score was significantly higher when animals underwent tattooing without prior application of EMLA compared to tattooing with prior application of EMLA and sham tattooing with and without prior application of EMLA (P = 0.001, P = 0.018, P = 0.002 respectively), with no other significant differences between treatments (see Figure 5). Only tattooing without prior application of EMLA was associated with a significant increase in the RbtGS score from pre to intra procedure (P<0.001), with no difference between the pre and intra procedure RbtGS scores for any of the other treatments (see Figure 5). Example images and associated RbtGS scores of rabbits in the tattoo and sham tattoo groups without prior application of EMLA are shown in Figure 6.

The average accuracy of global pain assessment was 83.6%, with misses being slightly more prevalent (10.6%) than false positives (5.8%). Individual accuracy varied from 62.5% to 86.5%. The Rabbit Grimace Scale also demonstrated high inter-rater reliability with an overall intraclass correlation coefficient (ICC) value of 0.91. All of the comprising facial action units also showed high ICC values; 0.94 for orbital tightening, 0.86 for cheek flattening and 0.84 for pointed nose.

### Home Cage Behaviour

Treatment had a significant effect on the duration of grooming (P = 0.034) and movement around the home pen (P = 0.026), and the frequency of rearing (P = 0.015). Treatment showed no significant influence on any of the other behaviours scored including previously validated pain behaviours (flinch, twitch, etc. [22], [23]) or shaking of the body and head.

#### Grooming

At 1-hour post-treatment, the duration of grooming the head and ears was significantly longer than baseline following the sham tattooing with and without prior application of EMLA (P = 0.03 respectively), and tattooing without prior application of EMLA (P = 0.05), but not with the tattooing with prior application of EMLA. There were no significant differences between the treatment groups at baseline or 1 h post-treatment (see Table 1). Treatment had no significant effect on the grooming of any other part of the body.

**Table 1:** The duration of grooming and movement and the frequency of rearing (mean ±1SE) at baseline compared to 1 hour post each of the four procedures (sham tattoo with/without EMLA & Tattoo with/without EMLA).

Behaviour	Baseline	Sham tattoo withoutEMLA	Sham tattoo with EMLA	Tattoo without EMLA	Tattoo with EMLA
Groom	50.0±29.7	220.5±40.8	179.1±51.7	242.6±70.9	136.3±63.6
Movement	219.5±62.0	43.4±12.5	55.8±26.8	29.6±3.8	58.3±40.9
Rear	8.1±2.4	1.0±0.4	1.8±0.7	0.5±0.1	0.6±0.2

#### Movement

At 1-hour post-treatment, the duration of movements were significantly shorter than baseline following the sham tattooing with and without prior application of EMLA (P = 0.01, P = 0.038 respectively), and tattooing with and without prior application of EMLA (P = 0.013, P = 0.05 respectively) (see Table 1). There were no significant differences between the treatment groups at baseline or 1 h post-treatment.

#### Rearing

At 1-hour post-treatment, the frequency of rearing was significantly lower than baseline following sham tattooing with and without prior application of EMLA (P = 0.03 respectively), and tattooing with and without prior application of EMLA (P = 0.013, P = 0.012 respectively) (see Table 1). There were no significant differences between the treatment groups at baseline or 1 h post-treatment.

## Discussion

Application of a needle tattoo device without prior application of EMLA cream caused clear physiologic and behavioural changes during and immediately following the procedure, which were absent following sham tattoo application. Consistent with previous findings, acute increases in heart rate and blood pressure occurred indicating a short period of intense pain [2]. The transient nature of this response is also evident from the similar area-under-the curve values among treatments for heart rate, as well as systolic, mean and diastolic blood pressure, measured for 2 minutes following the procedure. In addition to cardiovascular responses, immediate changes in behaviour, specifically struggling to escape restraint and vocalisation, and changes in facial expression also provided clear evidence of significant pain during this procedure. Rabbits rarely, if ever, vocalise in response to minor pain or discomfort, for example venepuncture, venesection or placement of catheters in superficial vessels [6]. Application of EMLA cream prior to tattoo administration almost completely eliminated all of these responses.

Beyond the immediate post-procedure period, home pen behaviours and corticosterone responses were similar irrespective of whether the animal underwent a tattoo or sham procedure with or without EMLA cream. Pain may be expressed through general behaviours, such as decreases in activity [17], or changes specific to regional pain such as back arching, twitching, staggering and abdominal writhing following laparotomy [22], [23]. One might expect changes associated with pain of the ear to involve shaking of the head or ears, or changes in grooming of the affected ear, however ear grooming did not differ among treatment groups. The duration of ear grooming increased for most of the treatments likely reflecting the presence of residual cream as opposed to pain. Rabbits also demonstrated less overall movement and a lower frequency of rearing in all treatment groups, which may reflect natural diurnal variation in activity [17]. Because of the minimal changes and similarities among treatments, behaviour was not evaluated beyond 1 hour following the procedure.

Changes in serum corticosterone also provide evidence of the transient nature of the pain and stress response. While concentrations were initially higher than baseline at 15 to 30 minutes post-procedure, corticosterone concentrations declined rapidly to return to baseline between 30 minutes to 1 hour post-procedure with all treatments. EMLA cream application had minimal effect on corticosterone concentrations suggesting that tattooing does not induce greater changes than those caused by handling and restraint alone, which are integral to both the tattoo and sham tattoo procedures. Although, application of a needle tattoo was associated with greater increase in corticosterone serum concentrations initially (15 minutes post-procedure) compared to the sham procedure, this effect did not persistent beyond 30 minutes post-procedure. These changes are not unexpected as changes in stress hormones are routinely observed in animals undergoing other potentially stressful procedures such as handling and introduction to novel environments [24]. Although the pain produced by tattoo application was transient, and behavioural and corticosterone changes in the post-tattoo period were minimal, we cannot exclude the possibility that prolonged mild pain or discomfort persisted after the tattooing procedure. However, it is clear that ear tattooing causes marked immediate behavioural and physiological responses consistent with the production of transient and potentially severe pain, which can almost be completely prevented by application of local anaesthetic cream. The degree of vocalisation and struggling exhibited by all of the rabbits during the tattooing with local anaesthetic strongly suggests that the pain experienced during this procedure was potentially severe. Such reaction is considered to indicate acute pain [25], and was ranked as the most severe clinical signs of pain following orthopaedic surgery in this species [26].

Evaluation of facial expression is a relatively recently developed means of assessing pain in animals. The growing popularity of this method is likely due to our previously poor ability to recognise pain in many species [11]–[13] and the ability of facial scales to offer accurate and reliable cage-side pain assessment [19], [20]. To develop the Rabbit Grimace Scale (RbtGS), pre-procedure images from all treatments were compared to those following tattoo administration without prior application of EMLA cream because of the obvious pain associated with this treatment. Facial changes in rabbits appear to be similar to those previously identified in mice and rats, with subtle variations between species. The facial action units comprising the Mouse Grimace Scale (MGS) are orbital tightening, nose bulge, cheek bulge, ear position, and whisker change [19]. The Rat Grimace Scale (RGS) was based on these changes and is largely similar, however it was noted that the nose and cheeks tended to flatten and elongate when rats are experiencing pain in contrast to the bulging seen in painful mice [19], [20]. The RbtGS shares greater similarities with the RGS as rabbits display a cheek and nose bulge at rest, which become progressively pointed or flattened as the intensity of pain increases.

Evaluation of the RbtGS scores assigned by blinded evaluators determined that the overall accuracy is lower than that of the MGS (97%) [19], but equivalent to that of the RGS (82%) [20]. This is most likely due to the photographs of rabbit faces used in this study being of variable quality. This is further supported by the study of Langford et. al [19] which clearly demonstrated that the higher the quality of the images given to participants the more accurately they were able score them. Although the accuracy is lower than the MGS, the overall inter-class correlation coefficient (ICC) is similar to those of both the MGS (0.90) [19] and RGS (0.90) [20]. As with other grimace scales applied to humans and animals (e.g. rodents), the participants in this study gave images of the rabbits in a non-painful state (e.g. pre-procedure) low but not zero scores. Such low scores are inevitable when using a scale that is a composite of four or more individual action units (e.g. orbital tightening, nose shape, cheek flattening & whisker position). In a non-painful state these action units can observed occasionally in isolation at a low intensity (score of 1 rather than 2), for example if an image is taken of a rabbit as it ‘blinks’ then an observer may give orbital tightening a score of 1 or 2 but 0 for the other action units.

EMLA cream is the most widely used topical anaesthetic, with demonstrated analgesic efficacy in superficial procedures, as well more invasive procedures such as the excision of subcutaneous lesions and skin graft removal [6]–[10]. The degree of desensitization provided depends on the dermal barrier to drug absorption, the area of the body, and the duration of contact time [27]–[29]. Following 60 minutes of contact time, EMLA cream results in acceptable analgesia to a mean depth of 2.9 mm in people [30]. Because of the minimal thickness of the rabbit’s pinna and application of EMLA cream to both sides of the ear in the current study, full thickness analgesia was achieved following 20 minutes of contact time. Additionally, skin on the rabbit pinna is more permeable than human skin [31] and this may have contributed to the rapid onset of analgesia. Although different contact times were not evaluated, it is possible a shorter application period could be equally effective in preventing pain associated with tattooing. The efficacy of EMLA cream for ear tattooing eliminates the need for general anaesthesia and may provide additional benefits such as on going pain relief. The analgesia provided by EMLA cream following a 30-minute application period continues for at least 180 minutes following removal in people [32] due to continued release of local anaesthetic from the skin ‘reservoir’. These findings may also be applicable to microchip insertion, ear tagging and ear notching in other species depending on the thickness of the tissue and contact time, and could represent a refinement that would improve the welfare of many species.

The ability of treatment-blinded evaluators to identify pain based on facial expressions in rabbits is in contrast to findings from a previous study using behaviour, which found that evaluators were less successful in correctly identifying pain in rabbits that had undergone ovariohysterectomy the more time they spent looking at the face [11]. This may be because changes can be subtle and easily missed without directed focus at specific facial changes, or because rabbits experiencing ongoing pain do not exhibit changes in facial expression to the same degree as following acute pain, possibly because they are a prey species and have evolved to mask signs of pain expression. Further studies are needed to determine whether rabbits display ‘pain faces’ in less acute pain states.

The findings from the current study clearly demonstrate the pain and distress caused by ear tattooing without analgesia, and that prior application of EMLA cream is effective in preventing almost all pain associated with this procedure. EMLA cream application can be considered a safe, effective, accessible method of analgesia that eliminates the need for general anaesthesia and can be used in non-veterinary environments. The Rabbit Grimace Scale has also been shown to correctly identify rabbits experiencing acute pain and may be a helpful tool when evaluating other husbandry or experimental procedures.

## Materials and Methods

### Ethical Statement

All procedures were carried out under project and personal licences approved by the Secretary of State for the Home Office, under the United Kingdom’s 1986 Animals (Scientific Procedures) Act and the Local Ethical Review Committee at Newcastle University. This study employed a strict ‘rescue’ analgesia policy. If any animal was deemed to be in greater then mild pain (assessed by an independent veterinarian), then buprenorphine (0.01 mg/kg *sc*) was immediately administered and the animal was removed from the study. Verbal informed consent was sought and gained from each participant prior to use of the rabbit grimace scale to score the rabbit images. This consent was simply documented by recording the number of participants who refused to take part. None of the participants approached refused to take part. Written consent was deemed unnecessary after consultation with Newcastle University. Verbal consent was deemed sufficient because all data was collected and analysed anonymously and no personal details of the participants were recorded. This study did not require institutional review board approval in order for it to be carried out.

### Animals and Husbandry

Eight barrier-reared New Zealand White Rabbits (4 male and 4 female) weighing 2–3 kg were obtained from a commercial supplier (Harlan, UK Ltd, Bicester, UK). Rabbits were housed singly in floor pens within sight, sound and touch of animals in adjacent pens, with males and females being housed in separate rooms. The rabbits were housed singly throughout the study to enable video footage and still images to be obtained and to prevent changes in behaviour or facial expressions resulting from transient separation from their cage mates. The animal room was maintained at 22±2°C, 50±10% humidity and on 12/12 h light/dark cycle. Food (Rabma pellets, SDS Ltd, Essex, UK) and tap water were provided *ad libitum* from a food hopper and water bottle attached to the pen wall. Each rabbit also received 300 g of fresh fruit and vegetables daily. Each pen contained autoclaved pine shavings and sawdust bedding at an average depth of 3 cm (Lillico Biotechnology, Bletchworth, UK), autoclaved hay (B&K Universal, Hull, UK), a plastic cat litter tray, a clean cardboard box, and a pine rabbit chew block (B&K Universal, Hull, UK). The animals were free from any common pathogens in accordance with the FELASA (Federation of Laboratory Animal Science Associations) health monitoring recommendations. Animals were allowed to acclimatise to the housing system for 14 days prior to the study beginning. During this time they were habituated to the general daily activity of the animal care staff, handling, weighing, the presence of the observer and the video monitoring equipment.

### Study Design and Treatments

A crossover design was employed, with the research team involved in data collection and application of the tattooing device being blinded to the treatments. Each rabbit underwent 4 treatments in semi-randomised order undergoing either an ear tattoo or a sham procedure, with either prior application of aqueous cream or EMLA cream. The tattoo was applied using handheld horizontal tattooing pliers (Haptner-Herberholz, GmBh and Co, Germany) loaded with 4×5 mm numbers, each consisting of 9 sharp pins of approximately 1 mm diameter that penetrated the full thickness of the ear, while the sham procedure consisted of application of the same device with a flat, unloaded plate. The rabbits underwent both tattoo treatments 7 days after the sham procedures were applied. This was to avoid hyperalgesia or allodynia, which could have been caused by tattooing, altering the responses to the sham procedure. Within each treatment block (sham or real tattoo) the sequence of prior application of EMLA or aqueous cream application and specific ear treated were randomized ensuring different ears were used for tattoo application with and without EMLA cream. A minimum ‘washout’ period of 2 days was implemented between treatment applications for each rabbit in each block. The only procedures that differed between the treatments was the application of a tattoo or not and the presence/absence of EMLA cream. All of the other common procedures (handling, restraint, catheterisation etc.) conducted on the rabbits in order to carry out the treatments remained constant.

### Procedure

Twenty minutes prior to a treatment, each rabbit was picked up and gently restrained in its home pen and a 1 mm thick layer of topical local anaesthetic cream (EMLA, Astra AstraZeneca, Luton, UK) or non-anaesthetic aqueous cream (E45 cream, Reckitt Benckiser Healthcare UK Ltd, UK) was applied to both inner and outer surfaces of the ear that was to be tattooed or handled (test ear) and covered with an occlusive dressing. EMLA cream was applied to the other ear, to prevent pain during vessel catheterisation. Application of either cream was carried out by a member of the research team who was not involved in any of the animal assessments to ensure that all other procedures were undertaken by treatment-blinded observers. After 15 minutes each animal was transferred to a procedure room, placed onto a non-slip table and gently restrained, the bandages and cream removed from the ears and 22G “over-the-needle” catheters (Abbocath, Abbott, Maidenhead, UK) placed in the ear vein and artery of the non-treated ear for blood sampling and direct measurement of arterial pressure. Adhesive ECG electrodes were attached to the forelimbs and left hindlimb. Rabbits were connected to a multi-parameter monitor (Kontron UK, Chichester, UK) to allow recording of the ECG and arterial blood pressure. Three high definition video cameras (Sony High Definition HandyCam model HDR-XR155, Sony, Japan) were positioned to allow recording of the monitor screen, the rabbit face and head, and the behavioural responses of the rabbit. Five minutes after being brought into the procedure room each rabbit underwent one of the 4 treatments: either ear tattooing or the sham procedure, with prior application of either aqueous cream or EMLA cream. The same operator carried out all treatments in all animals with the tattoo pliers being applied for 3 seconds in all treatments. Following each procedure the rabbit was then returned to its home pen for further data collection. No intra-procedure complications were reported and all rabbits recovered uneventfully.

### Physiological and Behavioural Recording

The immediate physiological response to the treatments was assessed by recording systolic, diastolic and mean blood pressure (mmHg) every 10 seconds for 120 seconds before and after treatment administration using a multi-parameter monitor (Kontron UK, Chichester, UK). The immediate behavioural reaction to the treatments was assessed by recording the frequency of struggling and vocalisation observed during the application of each treatment The assessment was made by a treatment blind observer from the video recordings taken using an ethogram of rabbit behaviour (see Table 2).

**Table 2:** Ethogram of rabbit behaviour (adapted from Leach et al. [17]).

Behaviour	Description
Reaction:	
Struggle	Struggling against gentle restraint during handling and procedures
Vocalisation	Emitting a audible sound during handling and procedures
Groom:	
Unaffected ear	The ear that has not undergone a treatment
Affected ear	The ear that has undergone a treatment
Head/face	Head or face using front or hind feet
Body & limbs	Any other part of the body by licking or scratching
Unknown	Unable to identify where due to rabbits position
Groom overall	All of the above behaviours
Activity:	
Movement	Movement around the pen by hopping, jumping or walking
Out of sight	Not visible due to hiding behind or under objects in pen
Head shake	Shaking the head from side-to-side
Body shake	Shaking of entire body from side-to-side
Object interact	Interaction with objects in the floor pen
Digging	Digging in the pen substrate
Eat/Drink	Eating and/or drinking
Stretch	Stretching of the body in various ways
Other	Behaviour not listed in ethogram
Inactive	Time spent completely motionless and inactive
Pain:	
Twitching	Rapid movement of fur on back
Flinching	Body jerks upwards for no apparent reason.
Wincing	Rapid movement of the backwards in a rocking motion accompanied by eye closing and swallowing action
Staggering	Partial loss of balance
Falling	Complete loss of balance when moving
Pressing	Abdomen pushed towards floor, usually before walking
Pain	Undefined behaviour (not observed in any other individual) that is believed to indicate pain
Arching	Full arching of the back upwards
Writhing	Contraction of the oblique flank muscles
Ear position:	
Flat	Ears flat on the back.
Erect	Ears erect and pointing in a specific direction.
Relaxed	Ears positioned between ‘flat’ and ‘alert’
Eye status:	
Open	Eyes wide open
Closed	Eyes tightly closed
Slit	Eyes semi-closed
Not visible	Eyes not visible due to rabbits position
Postures	
Crouch/sit	Sitting relaxed with hind limbs tucked under the rump and fore limbs underneath
Lie down	Lying down either with legs tucked under body or on right/left side of body
Rear	Rearing up with placement of fore limbs with or without support the wall of the pen or other object
Stand	Standing up on all four feet with abdomen off the floor

Plasma corticosterone concentrations were determined from blood samples taken the day prior to the study beginning (baseline) by venepuncture and at 15 minutes, 30 minutes, 1 hour, 2 hours and 3 hours post-treatment from the catheter in the non-treated ear. The venepuncture for baseline blood sampling involved each rabbit being picked up and gently restrained by an assistant. Each sample was taken within 3 minutes of the operators entering the floor pen. The corticosterone concentrations were assessed using a competitive enzyme-immunoassay (EIA) kit (IDS Plc, Boldon, UK).

Home cage behaviour was assessed from video recordings taken in the home pen. Fifteen-minute recordings were made on the day prior to treatment (baseline) and then at 1 hour post-treatment using a High definition video camera (Sony High Definition HandyCam model HDR-XR155, Sony, Japan). The camera was placed at a fixed distance from the front of the pen. This arrangement gave the highest probability of capturing the behaviour and face of the rabbit during filming. The behaviour observed in each video sequence (10 min epoch: same start time used for all clips) was scored using Observer XT (Version 9: Noldus Information Technology, Wageningen, Netherlands) according to an ethogram developed for assessing post ovariohysterectomy pain in rabbits [17] (see Table 2). This software was used to calculate the frequency and duration of the behaviours that were recorded.

### Rabbit Grimace Scale (RbtGS) Recording

**Figure 7:**
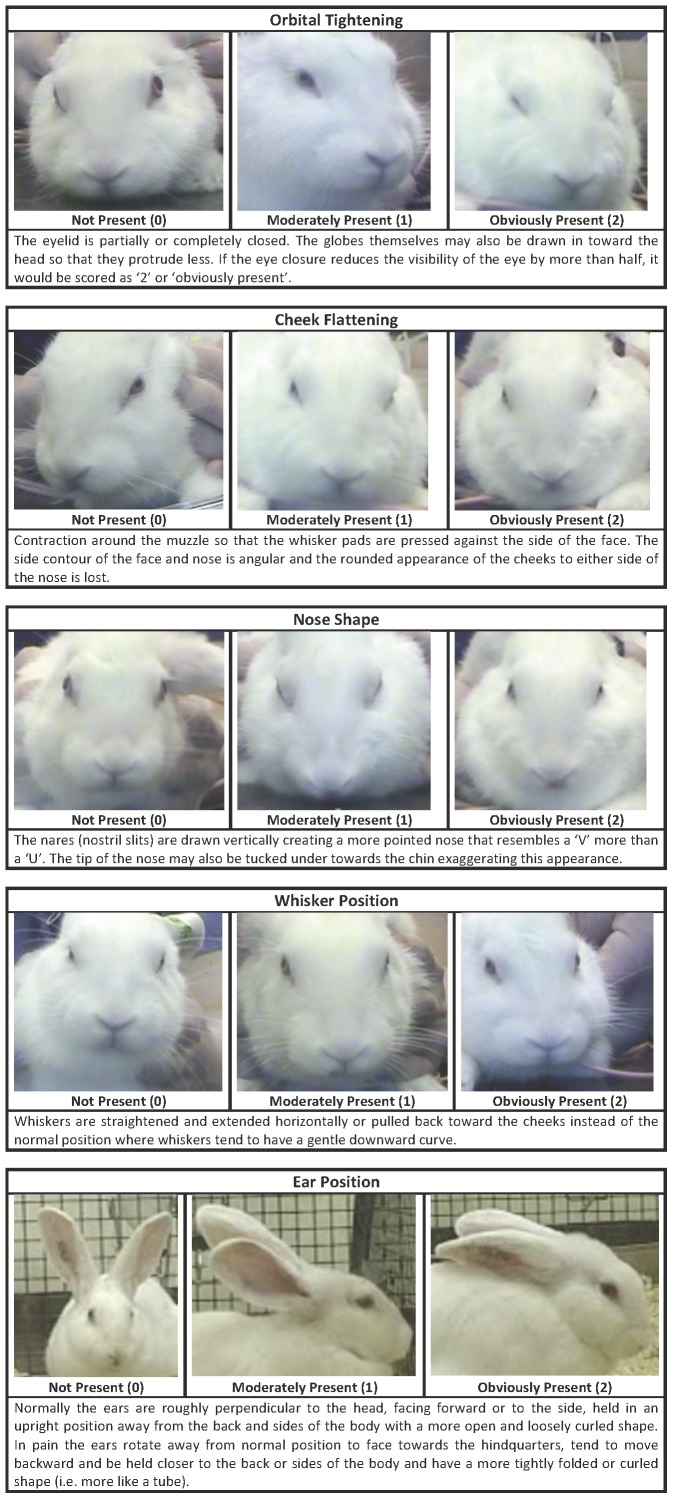
Rabbit Grimace Scale (RbtGS). The Rabbit Grimace Scale with images and explanations for each of the 5 facial action units (FAU); orbital tightening, cheek flattening, nose shape, whisker position and ear position. Each FAU is scored according to whether it is not present (score of 0), moderately present (score of 1) and obliviously present (score of 2).

The RbtGS was developed using the methods developed by Langford et al. [19] and Sotocinal et al. [20] for rodents by the investigators who were blind to the source of the photos (treatment & session) during development. From each video sequence of the rabbits’ head and face, still images were extracted, enabling generation of a number of clear and high quality images of each rabbit before and during each treatment. Each image was cropped so that only the head and not the body of the rabbit were visible. This prevented the observers from being biased by the body of the animal when attempting to score facial expressions [19]. Images taken before and during each procedure were compared to identify changes in facial expressions associated with these procedures by two-trained treatment and session blind observers experienced in assessing facial expressions in other species (MCL & SCJK). Based on these comparisons, the Rabbit Grimace Scale (RbtGS) was developed and is composed of five facial action units (FAUs); orbital tightening, cheek flattening, nose shape, whisker position and ear position (see Figure 7). From the extracted images 64 were selected at random by a non-participating assistant for further scoring and comprised 32 pre and 32 intra procedure images. Each of these two sets comprised one image pre procedure and one intra procedure image of each rabbit per treatment. The 64 images were then scored in a random order using the Rabbit Grimace Scale by ten treatment and session (pre or intra procedure) blind participants. Briefly, each participant was given a description and a pictorial guide (see Figure 7) of each of four Facial Action Units (FAUs); orbital tightening, nose shape, cheek flattening and whisker position. Ear position was not used as ears were manipulated during treatments and therefore their position was not representative. They were then asked for each image to give a score for each of FAU using a 3-point scale (0 = not present, 1 = moderately present & 2 = obviously present). If the participant was unable to see a particular FAU clearly, they were asked not to score it and to state that they could not determine it. The maximum score possible was 8 (i.e. a score of 2 for each of the 4 FAUs).

In order to explore the effect of time (pre vs. intra procedure) and treatment (sham tattoo vs. real tattoo with and without EMLA), the mean RbtGS scores were calculated for each image across all participants. The Rabbit Grimace Scale score for each image was determined by summing the score provided for each FAU with the exception of whisker position. Whisker position was excluded, as the majority of assessors were unable to score whisker position for many of the images, as they were not of high enough quality for whisker position to be clearly seen.

### Participant Selection

A total of 10 observers participated in this study and were recruited and tested in 2011 at Newcastle University. The observers were from diverse backgrounds and included veterinary surgeons, veterinary nurses, research scientists, animal technicians, psychology students and non-animal related occupations.

### Data and Statistical Analysis

All statistical analyses were conducted using SPSS 18 (SPSS Inc., Chicago, USA). The data were normally distributed with homogeneity of variance, so parametric analyses were carried out. Differences were considered to be statistically significant if P<0.05. A Chi-square analysis was used to compare the number of rabbits struggling and vocalising during the application of the various treatments. Repeated measures General Linear Models (GLM) were used to analyse the rabbit grimace scale and serum corticosterone concentration data with the time points (pre, intra & post-procedure), treatment group and treatment order as the within-subjects factors. Any time*treatment interactions were further investigated using repeated measures analysis of variance. In addition for the rabbit grimace scale, the change from pre to post procedure for each treatment group was calculated and compared to zero using a 1-sample t-test. The reliability of the scale was determined using inter-class correlation coefficient to compare mean scores for each of the facial action units across all of the participants. Accuracy was determined by comparing the global pain or no pain judgement made by the participants with actual pain state of the rabbit in each photograph (e.g. sham or actual tattoo with or without EMLA) as scored by treatment and session blind observer (MCL) with considerable experience in scoring post-procedural pain in rabbits using RbtGS and other measures. Repeated measures ANOVAs were used to analyse the change from pre to post-procedure and the area-under-curve for systolic arterial pressure (SAP), mean arterial pressure (MAP) and diastolic arterial pressure (DAP) and home case behaviour with treatment as the within subjects factor. All post-hoc analysis for the repeated measures ANOVA and GLM was conducted using a comparison of main effects with a Bonferroni interval adjustment.
